# Renal blood flow in sepsis

**DOI:** 10.1186/cc3540

**Published:** 2005-05-24

**Authors:** Christoph Langenberg, Rinaldo Bellomo, Clive May, Li Wan, Moritoki Egi, Stanislao Morgera

**Affiliations:** 1 Research fellow, Department of Intensive Care and Department of Medicine, Austin Hospital, and University of Melbourne, Heidelberg, Melbourne, Australia; 2 Director of Intensive Care Research, Department of Intensive Care and Department of Medicine, Austin Hospital, and University of Melbourne, Heidelberg, Melbourne, Australia; 3 Senior Researcher, Howard Florey Institute, University of Melbourne, Parkville, Melbourne, Australia; 4 Consultant Nephrologist, Department of Nephrology, Charité Campus Mitte, Berlin, Germany

## Abstract

**Introduction:**

To assess changes in renal blood flow (RBF) in human and experimental sepsis, and to identify determinants of RBF.

**Method:**

Using specific search terms we systematically interrogated two electronic reference libraries to identify experimental and human studies of sepsis and septic acute renal failure in which RBF was measured. In the retrieved studies, we assessed the influence of various factors on RBF during sepsis using statistical methods.

**Results:**

We found no human studies in which RBF was measured with suitably accurate direct methods. Where it was measured in humans with sepsis, however, RBF was increased compared with normal. Of the 159 animal studies identified, 99 reported decreased RBF and 60 reported unchanged or increased RBF. The size of animal, technique of measurement, duration of measurement, method of induction of sepsis, and fluid administration had no effect on RBF. In contrast, on univariate analysis, state of consciousness of animals (*P *= 0.005), recovery after surgery (*P *< 0.001), haemodynamic pattern (hypodynamic or hyperdynamic state; *P *< 0.001) and cardiac output (*P *< 0.001) influenced RBF. However, multivariate analysis showed that only cardiac output remained an independent determinant of RBF (*P *< 0.001).

**Conclusion:**

The impact of sepsis on RBF in humans is unknown. In experimental sepsis, RBF was reported to be decreased in two-thirds of studies (62 %) and unchanged or increased in one-third (38%). On univariate analysis, several factors not directly related to sepsis appear to influence RBF. However, multivariate analysis suggests that cardiac output has a dominant effect on RBF during sepsis, such that, in the presence of a decreased cardiac output, RBF is typically decreased, whereas in the presence of a preserved or increased cardiac output RBF is typically maintained or increased.

## Introduction

Acute renal failure (ARF) affects 5–7% of all hospitalized patients [1-3]. Sepsis and, in particular, septic shock are important risk factors for ARF in wards and remain the most important triggers for ARF in the intensive care unit (ICU) [4-8]. Among septic patients, the incidence of ARF is up to 51% [9] and that of severe ARF (i.e. ARF leading to the application of acute renal replacement therapy) is 5% [7,10]. The mortality rate associated with severe ARF in the ICU setting remains high [2-5,11].

A possible explanation for the high incidence and poor outcome of septic ARF relates to the lack of specific therapies. This, in turn, relates to our poor understanding of its pathogenesis. Nonetheless, a decrease in renal blood flow (RBF), causing renal ischaemia, has been proposed as central to the pathogenesis of septic ARF [12-14]. However, the bulk of knowledge about RBF in sepsis is derived from animal studies using a variety of different models and techniques. This creates uncertainty regarding the applicability of these studies to humans. Furthermore, the findings of studies in which experimental sepsis was induced and RBF measured have not been systematically assessed. Accordingly, we obtained all electronically identifiable publications reporting RBF in sepsis and analyzed the data according to changes in RBF. We also studied the possible influences of several technical and model-related variables on RBF.

## Materials and methods

We conducted a systematic interrogation of the literature using a standardized approach as described by Doig and Simpson [15] and Piper and coworkers [16]. We used two electronic reference libraries (Medline and PubMed), and searched for relevant articles using the following search terms: 'renal blood flow', 'kidney blood flow', 'renal blood supply', 'kidney blood supply', 'organ blood flow', 'organ blood supply', 'sepsis', 'septic shock', 'septicemia', 'caecal puncture ligation', 'cecum puncture ligation', 'lipopolysaccharide' and 'endotoxin'. We selected all animal studies published in the English language literature. Using the reference lists from each article, we identified and obtained other possible studies that might have reported information on RBF in septic ARF and that had not been identified by our electronic search strategy.

We assessed all human articles in detail. Because of the heterogeneity animal studies and the methods they employed, we also assessed all animal articles systematically for information on variables that might have influenced RBF in sepsis. The variables of interest were as follows: size of animal; technique of measurement for RBF (direct measurement via flow probe or microsphere technique or other technique); consciousness of animals during the study; recovery period between preparation surgery and the experiment; timing of RBF measurement in relation to septic insult; method used to induce sepsis (lipopolysaccharide [LPS], live bacteria, or caecal ligation–perforation technique); fluid administration during the experiment; cardiac output (CO); and haemodynamic patterns (hypodynamic and hyperdynamic sepsis).

Information obtained on RBF from these groups was compared. Comparisons were performed using the ?^2 ^test or Fisher exact test where appropriate. Variables were also entered into a multivariate logistic regression analysis (MVLRA) model with RBF as the dependent variable. *P *< 0.05 was considered statistically significant.

## Results

### Human studies

We found only three studies conducted in septic ICU patients in which RBF was measured [17-19]. The findings of these studies suggest an increase in RBF during sepsis (Table 1). In only one patient was renal plasma flow (RPF) determined in the setting of oliguric ARF [19]. Such RPF was markedly increased at 2000 ml/min (normal 650 ml/min).

### Animal models

**Figure 1:**
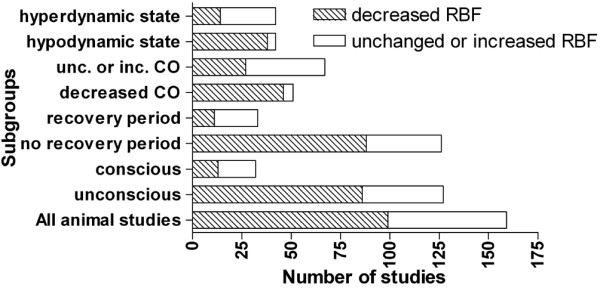
Effect of variables on renal blood flow: statistically significant findings. All of the differences between the shaded areas are statistically significant (*P *< 0.05). CO, cardiac output; inc, increased; RBF, renal blood flow; unc, unchanged.

We found 159 [20-178] animal studies that measured RBF in sepsis. Of these, 99 (62%) reported a decrease, whereas the remaining 60 (38%) studies reported no change or an increase in RBF (Table 2, Fig. 1).

#### Animal size

**Figure 2:**
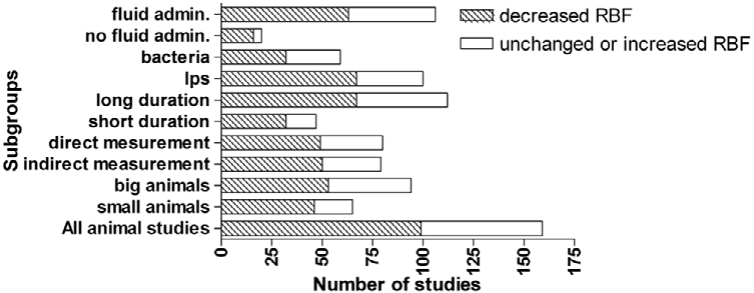
Effect of variables on renal blood flow: nonsignificant findings. None of the differences between and shaded areas are statistically significant. lps, lipopolysaccharide.

Experimental studies were conducted in a large variety of animals. We divided experimental animals into small (rats, mice, rabbits and piglets) and large (dogs, pigs and sheep). We identified 65 (41%) studies that were conducted in small animals and 94 (59%) that were conducted in large animals (Table 2). Of studies conducted in small animals, 46 found decreased and 19 (29%) unchanged or increased RBF. In large animals, 53 (56%) studies reported a decreased and 41 (44%) an unchanged or increased RBF (*P *= 0.066; Fig. 2).

#### Technique for measuring renal blood flow

The techniques used for the measurement of RBF varied widely. Therefore, we compared studies using direct measurement of RBF via ultrasonic or electromagnetic flow probes ('direct' techniques) with measurement by microsphere technique or para-aminohippurate (PAH) clearance or other techniques such as measurement of blood velocity via video microscopy ('indirect' techniques). Of 80 studies using flow probes, 49 (61%) showed a decreased and 31 (39%) an unchanged or increased RBF (Table 2). Of 79 studies using other methods, 50 (63%) reported a decreased and 29 (37%) reported an unchanged or increased RBF (*P *= 0.791; Table 2, Fig. 2).

#### Consciousness of animals

The use of awake or unconscious animals might also have influenced RBF. For this reason, we compared studies using conscious with those using unconscious animals. Of 127 experiments conducted in unconscious animals (Table 2), 86 (68%) reported a decreased and 41 (32%) an unchanged or increased RBF. Of 32 studies conducted in conscious animals (Table 2), 13 (41 %) reported a decreased and 19 (59%) reported no change or an increase in RBF (*P *= 0.005; Fig. 1).

#### Recovery period between surgical preparation and actual experiment

Before conducting the experiments, a surgical procedure is typically needed to prepare the animals. We compared studies starting the experiment immediately after surgery with studies with a recovery period after anaesthesia. Of 33 studies with a recovery period (Table 2), 11 (33%) showed a decreased and 22 (67%) showed an unchanged or increased RBF. Of 126 studies without a recovery period (Table 2), 88 (70%) reported a decreased and 38 (30%) reported no change or an increase in RBF (*P *< 0.001; Fig. 1).

#### Time from septic insult

The duration of RBF observation after the septic insult varied widely. We divided the studies into those with a 'short' (<2 hours; early period after induction of sepsis) or 'long' (>2 hours; late period after the induction of sepsis) observation time. Among 47 experiments with short periods of observation after the induction of sepsis (Table 2), 32 (68%) showed a decreased and 15 (32%) showed an unchanged or increased RBF. Among the 112 experiments with long periods of observation after the induction of sepsis (Table 2), 67 (60%) showed a decreased and 45 (40%) showed an unchanged or increased RBF (*P *= 0.327; Fig. 2).

#### Methods of inducing sepsis

Many different methods of induction of sepsis were used. We compared LPS-induced sepsis with sepsis induced by injection of live bacteria or caecal ligation–perforation. Of 100 articles that used LPS (Table 2), 67 (67%) showed a decreased and 33 (33%) showed an unchanged or increased RBF. Among the other 59 studies (Table 2), 32 (54%) reported a reduced and 27 (46%) reported an unchanged or increased RBF (*P *= 0.109; Fig. 2).

#### Fluid administration

We compared studies according to whether there was fluid administration during the experiments. Thirty-four articles did not mention fluid administration. Among the 20 studies with no fluid administration (Table 2), 16 (80%) reported a decreased and 4 (20%) reported an unchanged or increased RBF. Of the 106 studies in which fluid was given (Table 2), 63 (59%) showed a decrease and 43 (41%) showed no change or an increase in RBF (*P *= 0.081; Fig. 2).

#### Haemodynamic patterns

Most septic patients exhibit a hyperdynamic state with elevated CO and decreased blood pressure, when CO is measured. Therefore, we compared studies in which animals had a hyperdynamic state (low peripheral vascular resistance [PVR]) of sepsis with studies in which this state was not present (normal or high PVR). There were 84 studies in which the hypodynamic versus hyperdynamic pattern could be assessed. Of 42 studies that fulfilled criteria for hypodynamic sepsis (Table 2), 38 (90%) showed a reduced and 4 (10%) showed no change or an increase in RBF. Of the 42 studies conducted in hyperdynamic sepsis (Table 2), 14 (33%) reported a decreased and 28 (67%) reported an unchanged or increased RBF (*P *< 0.001; Fig. 1).

#### Cardiac output

We compared those studies with increased or unchanged CO with studies with decreased CO. Some studies gave no indication of CO. Of the 51 studies with decreased CO (Table 2), 46 (90%) reported a decreased and 5 (10%) reported an unchanged or increased RBF. Among the 67 studies with an unchanged or increased CO (Table 2), 27 (40%) showed a reduced and 45 (60%) showed an unchanged or increased RBF (*P *< 0.001; Fig. 1).

Using MVLRA, we created a model to test for independent determinants of a RBF and found that only CO remained in the model (*P *< 0.001) as a significant predictor for RBF (Table 3).

## Discussion

We interrogated two electronic databases to assess the changes that occur in RBF during human and experimental sepsis in order to examine what might be the determinants of sepsis-associated changes in RBF. Variables that might influence RBF were used to categorize the heterogeneous data we found.

We found only a few human studies reporting RBF in a septic setting and found that the techniques used to measure RBF had poor accuracy and reproducibility. Only in a single patient with septic oliguric ARF was RBF measured. Nonetheless, within these serious limitations, we found that an increase in RBF was typically seen during sepsis.

We found that most animal studies reported a decrease in RBF in sepsis. However, we found that, in one-third of studies, RBF was either maintained or increased. We also found contradictory and inconsistent experimental findings with regard to RBF, which appeared to be affected by factors other than the induction of sepsis itself, including the consciousness of the animal, the recovery time after surgery and the haemodynamic pattern (hypodynamic or hyperdynamic state). More importantly, using MVLRA, we found that all of the above factors could be reduced to the dominant effect of CO on RBF. Thus, a low CO predicted a decreased RBF and a preserved or high CO predicted an unchanged or increased RBF. These findings are complex and require detailed discussion.

### Human studies

Currently, only invasive techniques for measuring RBF have a high degree of accuracy. They require renal vein sampling. Because of the risks associated with such invasive measurement of RBF, only a few such studies have been conducted in humans with sepsis. Noninvasive methods of measurement such as the PAH clearance method are also possible but they assume a constant PAH extraction ratio of 0.91, such that RPF can be calculated with measurement of PAH concentrations in blood and urine. Unfortunately, the 'constant' PAH extraction ratio is not at all constant, is markedly unstable and is influenced by many factors, all of which apply in sepsis and ARF [18,19]. Therefore, in order to achieve improved accuracy, this method must be made invasive by inserting a renal vein catheter in order to calculate the true PAH extraction ratio. The RPF measured by this method is called the true RPF. Finally, a third method uses a thermodilution renal vein catheter. RPF and RBF determined by the thermodilution method were reported to correlate with corrected PAH clearances (r = 0.79) [17].

However, a recently reported study [179] demonstrated that both methods have a low reproducibility and a within group error of up to 40%. Therefore, these methods are not sufficiently accurate to detect potentially important changes in RBF. Nonetheless, within the boundaries of the technology, true RPF measurements from human studies (Table 1) consistently suggest that renal blood flow is *increased *during human sepsis. In only one study [19] was RBF estimated in a septic patient with ARF. The RPF was found to be 2000 ml/min in this patient, which contrasts with the normal RPF in humans of 600–700 ml/min [180].

### Animal models

#### Animal size

In small animals, RBFs values are very small (7.39 ml/min [40]). The changes estimated in different settings are even smaller (1.4 ml/min [40]). On the other hand, absolute blood flows in large animals are up to 250 times greater (330 ml/min [55]). We hypothesized that measurement accuracy might therefore change with animal size and lead to different observations. We found a strong trend in this direction, which just failed to achieve statistical significance.

#### Technique of measurement if renal blood flow

Using the flow probe technique, it is possible to measure the RBF continuously. Microsphere techniques are also accurate and can distinguish between cortical and medullar RBF, but using the latter technique it is only possible to take several 'snapshot views' of blood flow during the experiment. We hypothesized that the technique of measurement might have influenced findings. However, there was no significant difference between techniques.

#### Consciousness of animals

Most studies were conducted in unconscious animals. Within this group, RBF was significantly more likely to be decreased than in conscious animals. This effect might partly be explained by anaesthesia rather than sepsis itself. Our observations highlight this as an important area of concern in drawing conclusions about the effect of sepsis *per se *on RBF.

#### Time from septic insult

A recently published animal study [55] described the time-dependent development of hyperdynamic sepsis after live *Escherichia coli *injection. In that study the CO decreased immediately after injection, recovered and then increased by 2 hours until a hyperdynamic state was reached. Therefore, we divided the studies in experiments with less or greater than 2 hours of observation time after the septic insult in order to determine whether there were differences between early and late septic states. We hypothesized that studies with longer periods of observation after the insult (late sepsis) might show a different RBF. However, there was no difference between the two groups.

#### Recovery period

Surgical preparation was performed in many of the reviewed studies just before the experiments were started. The negative effect on RBF of immediately beginning the experiments after surgery might be explained by the prolonged anaesthesia time and the negative effect of anaesthesia. We found that lack of an adequate recovery period after surgical preparation increased the likelihood of RBF being decreased.

#### Method of inducing sepsis

Many different techniques are used to induce sepsis such as LPS injection, live bacteria injection and caecal ligation–perforation. Previous reports [181,182] described a strong hypodynamic effect of injecting a bolus of LPS. Therefore, we hypothesized that studies using LPS might show decreased RBF. We found a trend in this direction that approached statistical significance.

#### Fluid administration

Most of the studies administered fluid during the experiments to counteract the hypotensive of effect of sepsis [14]. These fluids might maintain CO, central venous pressure and blood pressure, and thus affect RBF. As might be expected, we found a strong trend toward a higher RBF when fluid resuscitation was given, but this failed to achieve statistical significance.

#### Haemodynamic patterns

In septic patients, CO, blood pressure and PVR can be assessed. Most of these patients have an increased CO, a low blood pressure and a decreased PVR [14,183-189]. To assess what might happen to RBF in a haemodynamic situation simulating human sepsis, we compared studies with animals that had developed hyperdynamic sepsis (increased CO and decreased blood pressure) with those studies with hypodynamic sepsis (normal or increased PVR). Animals with hyperdynamic sepsis were more likely to exhibit preserved or increased RBF.

#### Cardiac output

In a recently published article [190] using a crossover animal model, CO was found to be the most important variable influencing organ blood flows. Thus, we compared studies showing an unchanged or increased CO with studies showing a decreased CO. We found a clear association between decreased CO and decreased RBF and between a preserved or increased CO and a preserved or increased RBF. Multivariate logistic analysis confirmed the role of CO as the most powerful independent predictor of RBF in sepsis (Table 2).

### Limitations

We only interrogated two English language electronic reference libraries and might have missed original contributions reported in other languages. However, we believe that it is unlikely that enough such studies would exist to change our conclusions materially.

In order to make comparisons, we categorized experiments according to pre-set criteria (small versus large animals, methods of induction of sepsis, high versus low CO, etc.) that we hypothesized, on grounds of biological plausibility, were likely to affect experimental findings. We acknowledge that such criteria are by definition arbitrary and the subject of individual judgement. Furthermore, other criteria that we did not consider could be tested. Nonetheless, we found that many of these criteria appeared to have some effect in reality. We also found that such effects appeared to be mostly related to their association with the CO state, which overwhelmingly was the only independent predictor in MVLRA for the outcome of RBF. We consider it unlikely that the choice of other criteria for comparison would materially affect our conclusions.

The observation time in the reviewed articles varied widely as well. We compared articles with a shorter period after the insult (2 hours) versus studies with a longer period of observation. We acknowledge that this division is artificial and might not truly reflect what happened, because some groups waited until the animal reached defined criteria before starting their observation time and others begun the observation immediately after the septic insult, making this variable extremely heterogeneous. Nonetheless, once again, given the overwhelming effect of CO on RBF, we consider that refinements to this criterion are unlikely to influence our conclusions.

Our observations suggest that the widely held paradigm that RBF decreases in sepsis [12-14] and that such a decrease is responsible for the development of ARF is indeed sustained by the majority of studies. However, the reality beyond such a simplistic observation is much more complex. The animal studies are extraordinarily heterogeneous in their design and monitoring of RBF. Furthermore, the support that the bulk of the data offer to the concept of decreased RBF in sepsis is conditional upon a particular model of sepsis being present (hypodynamic sepsis without an increase in CO). If the CO is increased and PVR is decreased, then the most common finding is actually one of increased or preserved RBF. In the light of this review, we suggest that measurement of CO is a vital component of all future experimental studies measuring RBF in sepsis.

We note that, in human sepsis, systemic vasodilatation with a high CO is the dominant clinical finding. Such vasodilatation might also affect the afferent and efferent arterioles of the kidney. If the efferent arteriole dilated proportionately more than the afferent arteriole, then there would be a decrease in glomerular filtration pressure. This change in filtration pressure would decrease glomerular filtration rate and lead to oliguria and loss of small solute clearance. Accordingly, loss of glomerular filtration rate can occur with either vasoconstriction or vasodilatation.

Our findings have important implications for clinicians and for future strategies directed at preserving renal function in sepsis. They highlight the absence of human data. They show the heterogeneity and model dependence of the animal data. They also emphasize the limitations of the indirect data upon which clinical strategies are based. Much research remains to be done if we are to establish what happens to renal blood flow in human sepsis, and techniques are needed that permit such measurements to be taken noninvasively.

## Conclusion

We interrogated the two major English language electronic reference libraries to examine changes in RBF in sepsis and septic ARF. We found that inadequate data exist to allow any conclusions to be drawn on the typical RBF or changes in RBF in humans. We also found that experimental data are extraordinarily heterogeneous in nature but show the dominant effect of CO on RBF, such that a low CO predicts a decreased RBF and an increased or preserved CO predicts an increased or preserved RBF. Given that CO is typically increased when measured in human sepsis in the ICU, the widely held paradigm that decreased RBF is pivotal to the pathogenesis of septic ARF might require reassessment.

## Key messages

• 	It is unknown whether RBF is increased, decreased, or unchanged in human sepsis.

• 	Techniques to measure RBF in humans are invasive and of limited accuracy.

• 	Data on RBF from animals are heterogenous and do not allow firm conclusions to be drawn.

• 	RBF findings in experimental sepsis depend on the model used.

• 	CO is the most important independent predictor of RBF in sepsis: if CO is increased, then RBF is typically increased; and if CO is decreased, the RBF is typically decreased.

## Abbreviations

ARF = acute renal failure; CO = cardiac output; ICU = intensive care unit; LPS = lipopolysaccharide; MVLRA = multivariate logistic regression analysis; PAH = para-aminohippurate; PVR = peripheral vascular resistance; RBF = renal blood flow; RPF = renal plasma flow.

## Competing interests

The author(s) declare that they have no competing interests.

## Authors' contributions

CL conducted the searches and reviewed all necessary material, wrote the initial draft of the manuscript and performed statistical analysis. RB designed the study, critically reviewed the material and supervised the writing of the manuscript, CM co-designed the study and assisted with the completion of the manuscript. LW assisted with data assessment. ME assisted with data assessment and statistical analysis. SM assisted with study design and assessment, and completion of the manuscript.

## Figures and Tables

**Table 1:** Details of human studies conducted in septic patients measuring renal blood flow

Reference	Measurement of PAH-RPF/true RPF (*n*/*n*)	PAH-RPF (ml/min)	True RPF (ml/min)
[17]	6 (0)	-	690
[18]	40 (11)	475	1116
[19]	22 (6)	474	1238

PAH-RPF, renal plasma flow calculated using para-aminohippurate clearance with no renal vein sampling; true RPF, true renal plasma flow (flow calculated with renal vein sampling for PAH).

**Table 2:** References for studies reporting various findings pertaining to RBF in experimental sepsis

Finding/study characteristic	Number of studies (%)	References
Decrease in RBF	99 (62%)	20, 21, 23, 24, 26-29, 37-45, 49-54, 58-64, 68-70, 73, 74, 76, 78, 80, 83-86, 88, 90-95, 98-101, 103-107, 109, 110, 112, 113, 118-121, 123, 124, 126, 128-131, 134, 135, 140, 143-145, 149, 150, 152-157, 159, 160, 163, 165, 168, 169, 171-175 and 178
No change or a decrease in RBF	60 (38%)	22, 25, 30-36, 46-48, 55-57, 65-67, 71, 72, 75, 77, 79, 81, 82, 87, 89, 96, 97, 102, 108, 111, 114-117, 122, 125, 127, 132, 133, 136-139, 141, 142, 146-148, 151, 158, 161, 162, 164, 166, 167, 170, 176 and 177
Conducted in small animals (rats, mice, rabbits and piglets)	65 (41%)	20, 24, 27, 28, 38, 40, 43-45, 49, 50, 59, 61, 62, 65-67, 71-74, 77, 78, 82, 87, 88, 90, 92, 93, 99, 100, 102-105, 109, 110, 112, 115, 116, 119-121, 126, 128, 133, 138, 139, 141, 145, 149, 150, 152, 155-157, 159, 160 and 164-170
Conducted in largane animals (dogs, pigs and sheep)	94 (59%)	21-23, 25, 26, 29-37, 39, 41, 42, 46-48, 51-58, 60, 63, 64, 68-70, 75, 76, 79-81, 83-86, 89, 91, 94-98, 101, 106-108, 111, 113, 114, 117, 118, 122-125, 127, 129-132, 134-137, 140, 142-144, 146-148, 151, 153, 154, 158, 161-163 and 171-178
Measurement of RBF using flow probes	80 (50%)	21, 23-26, 28, 29, 32, 33, 36, 42, 43, 46, 53-58, 60, 63, 65, 66, 68-70, 75, 79, 81-85, 89, 91, 92, 95, 98, 101, 104-108, 110, 111, 113, 115, 116, 118, 122, 124-127, 129, 131, 132, 134-136, 142-144, 148, 153, 158-163 and 171-178
Measurement of RBF using other methods	79 (50%)	20, 22, 27, 30, 31, 34, 35, 37-41, 44, 45, 47-52, 59, 61, 62, 64, 67, 71-74, 76-78, 80, 86-88, 90, 93, 94, 96, 97, 99, 100, 102, 103, 109, 112, 114, 117, 119-121, 123, 128, 130, 133, 137-141, 145-147, 149-152, 154-157 and 164-170
Conducted in unconscious animals	127 (80%)	21-29, 37-44, 46, 53, 54, 57, 60-63, 65-75, 77-89, 91-101, 103-107, 109-111, 113, 115, 116, 118-121, 123-136, 138-160, 163, 164 and 166-178
Conducted in conscious animals	32 (20%)	20, 30-36, 45, 47-52, 55, 56, 58, 59, 64, 76, 90, 102, 108, 112, 114, 117, 122, 137, 161, 162 and 165
Conducted following a recovery period (after surgical preparation)	33 (21%)	30-36, 47-52, 55-59, 64, 68, 70, 76, 102, 108, 112, 114, 117, 122, 137, 161, 162, 166 and 170
Conducted with no recovery period	126 (79%)	20-29, 37-46, 53, 54, 60-63, 65-67, 69, 71-75, 77-101, 103-107, 109-111, 113, 115, 116, 118-121, 123-136, 138-160, 163-165, 167-169 and 171-178
Short period of observation following induction of sepsis (<2 hours)	47 (29%)	22, 26, 27, 40, 41, 47, 49, 50, 57, 59-61, 67, 70, 79, 80, 82, 86, 89, 92, 99, 100, 103, 105, 106, 109, 111, 117, 120, 121, 123, 124, 129, 130, 145-147, 149-151, 153, 154, 156, 158, 163, 164 and 167
Long period of observation following induction of sepsis (>2 hours)	112 (71%)	20, 21, 23-25, 28-39, 42-46, 48, 51-56, 58, 62-66, 68, 69, 71-78, 81, 83-85, 87, 88, 90, 91, 93-98, 101, 102, 104, 107, 108, 110, 112-116, 118, 119, 122, 125-128, 131-144, 148, 152, 155, 157, 159-162, 165, 166 and 168-178
Use of LPS to induce sepsis	100 (63%)	21, 23-26, 28, 29, 37, 39, 40, 42-46, 50, 54, 58-61, 63, 65, 66, 68-72, 76, 79, 80, 82, 86-97, 101, 103-106, 109-111, 114-118, 120-127, 129-136, 141-144, 147-150, 153-158, 160-164, 171, 172 and 174-178
Use of injection of live bacteria or caecal ligation–perforation to induce sepsis	59 (37%)	20, 22, 27, 30-36, 38, 41, 47-49, 51-53, 55-57, 62, 64, 67, 73-75, 77, 78, 81, 83-85, 98-100, 102, 107, 108, 112, 113, 119, 128, 137-140, 145, 146, 151, 152, 159, 165-170 and 173
Fluid administered during the experiment^a^	20 (13%)	22, 27, 61, 68, 69, 72, 77, 78, 83, 85, 91, 113, 118, 121, 130, 135, 136, 144, 145 and 150
Fluid not administered during the experiment^a^	106 (67%)	21, 23-26, 28-32, 34-41, 43-46, 48-52, 54-59, 62-66, 71, 73-76, 79, 80, 82, 84, 87, 90, 92-101, 103-105, 107, 108, 111, 112, 114-116, 119, 122-129, 131, 137-140, 143, 146-148, 151-153, 155, 157-159, 161, 162, 165-167, 169, 170, 173-176 and 178
Conducted in hypodynamic sepsis^b^	42 (26%)	37, 39, 42-44, 53, 54, 58, 61, 63, 68, 69, 80, 84, 86, 89, 98, 101, 103, 107, 113, 118, 120, 121, 127, 129, 132, 140, 144, 149, 151, 154-157, 165, 172-174 and 178
Conducted in hyperdynamic sepsis^b^	42 (26%)	20, 26, 30-36, 41, 46-48, 51, 55-57, 76-79, 81, 83, 96, 97, 100, 102, 105, 111, 117, 122, 123, 125, 131, 150, 153, 158, 161, 162 and 175-177
Decreased CO^c^	51 (32%)	21, 25, 29, 37-39, 42-44, 53, 54, 58, 59, 61, 63, 68, 69, 80, 84, 86, 88, 89, 98, 101, 103, 107, 112, 113, 118, 120, 121, 127-130, 132, 140, 144, 149, 151, 154-157, 165, 168, 169, 172-174 and 178
Unchanged or decreased CO^c^	67 (42%)	20, 26, 27, 30-36, 40, 41, 46-52, 55-57, 64, 73, 74, 76-79, 81, 83, 90, 96, 97, 99, 100, 102, 105, 108, 111, 114, 117, 119, 122, 123, 125, 131, 133, 137-139, 141, 145, 148, 150, 152, 153, 158, 161, 162, 164, 166, 167, 170 and 175-177]

^a^Some studies did not mention fluid administration. ^b^It was not possible to assess in some studies whether a septic hyperdynamic versus hypodynamic state was present. ^c^Some studies gave no indication of CO. CO, cardiac output; LPS, lipopolysaccharide; RBF, renal blood flow.

**Table 3:** Multivariate logistic regression analysis of possible predictors of renal blood flow in experimental sepsis

Variable	Regression coefficient	95% confidence interval	*P*
Cardiac output	3.658	5.916–254.468	<0.001
Blood pressure	-0.796	0.076–2.669	0.380
Recovery period	2.767	0.340–745.908	0.159
Consciousness	-2.650	0.001–4.318	0.207
Fluid administration	2.066	0.543–114.722	0.130
Animal size	1.043	0.362–22.230	0.321
Technique measurement	0.608	0.390–8.666	0.442
Duration	1.496	0.849–23.482	0.077
Method of insult	0.501	0.374–7.284	0.508
